# Constructing a screening model to identify patients at high risk of hospital-acquired influenza on admission to hospital

**DOI:** 10.3389/fpubh.2025.1495794

**Published:** 2025-04-16

**Authors:** Shangshu Zhang, Peng Li, Bo Qiao, Hongying Qin, Zhenzhen Wu, Leilei Guo

**Affiliations:** ^1^Department of Disease Prevention and Control, Zhengzhou Central Hospital Affiliated to Zhengzhou University, Zhengzhou, Henan, China; ^2^Department of Hospital Infection Control, Henan Provincial People's Hospital, People's Hospital of Zhengzhou University, Zhengzhou, China; ^3^Department of Hospital Infection Control, Henan Provincial Chest Hospital, Zhengzhou University, Zhengzhou, China; ^4^Department of Infection Prevention and Control, Zhengzhou Central Hospital Affiliated to Zhengzhou University, Zhengzhou, Henan, China

**Keywords:** hospital-acquired influenza, machine learning, prediction model, SHAP (SHapley’s additive explanation), practical tool

## Abstract

**Objective:**

To develop a machine learning (ML)-based admission screening model for hospital-acquired (HA) influenza using routinely available data to support early clinical intervention.

**Methods:**

The study focused on hospitalized patients from January 2021 to May 2024. The case group consisted of patients with HA influenza, while the control group comprised non-HA influenza patients admitted to the same ward in the HA influenza unit within 2 weeks. The 953 subjects were divided into the training set and the validation set in a 7:3 ratio. Feature screening was performed using least absolute shrinkage and selection operator (LASSO) and the Boruta algorithm. Subsequently eight ML algorithms were applied to analyze and identify the optimal model using a 5-fold cross-validation methodology. And the area under the curve (AUC), area under the precision-recall curve (AP), F1 score, calibration curve and decision curve analysis (DCA) were applied to comprehensively assess the predictive effectiveness of the selected models. Feature factors were selected and feature importance’s were assessed using SHapley’s additive interpretation (SHAP). Furthermore, an interactive web-based platform was additionally developed to visualize and demonstrate the predictive model.

**Results:**

Age, pneumonia on admission, Chronic renal failure, Malignant tumor, hypoproteinemia, glucocorticoid use, admission to ICU, lymphopenia, BMI were identified as key variables. For the eight ML algorithms, ROC values ranging from 0.548 to 0.812 were observed in the validation set. A comprehensive analysis showed that the XGBoost model predicted the highest accuracy (AUC: 0.812) with an F1 score of 0.590 and the highest A *p* value (0.655). Evaluating the optimal model, the AUC values were 0.995, 0.826, and 0.781 for the training, validation and test sets. The XGBoost model showed strong robust. SHapley’s additive interpretation (SHAP) was utilized to analyze the contribution of explanatory variables to the model and their correlation with HA influenza. In addition, we developed a practical online prediction tool to calculate the risk of HA influenza occurrence.

**Conclusion:**

Based on the routine data, the XGBoost model demonstrated excellent calibration among all ML algorithms and accurately predicted the risk of HA influenza, thereby serving as an effective tool for early screening of HA influenza.

## Introduction

Influenza is one of four categories of respiratory infectious diseases with potential pandemic risk. There are one billion cases of seasonal influenza worldwide each year, and it is the leading cause of lower respiratory tract infections worldwide (1, 2). Influenza causes significant morbidity and mortality in the United States and has pandemic potential. The burden of influenza has been on the rise after the COVID-19 pandemic. The interim estimated burden of influenza for the 2023–2024 influenza season indicated that between 35 and 65 million illnesses, 390,000 and 830,000 hospitalizations, and 25,000 and 72,000 deaths occurred that season (3). Additionally, studies have revealed that there are an average of 88,100 excess influenza-associated respiratory disease deaths per year in China, accounting for 8.2% of respiratory disease deaths (4).

Hospital-acquired (HA) influenza has been shown to be associated with high mortality, leading to prolonged hospitalization and increased healthcare costs. Accumulating evidence showed that HA influenza may contribute to 11.38% of influenza cases (4)with mortality rates reaching as high as 18.8% (5) and severe illness incidence peaking at 39.2% (6). Furthermore, several studies have reported outbreaks of influenza in hospitals and in-ward transmission (7–9). HA influenzas represent the primary public health emergencies associated with hospital-acquired infections in China (10). However, current hospital infection surveillance systems primarily concentrate on detection of bacteria, overlooking the target monitoring of HA influenza and frequently underestimating the incidence of HA influenza. Therefore, the aim of this study was to promptly identify patients at high risk of HA influenza so as to lower the risk of nosocomial infection outbreaks and early implement specific intervention strategies to reduce the incidence of HA influenza.

Although there have been numerous studies on the epidemiological characteristics and risk factors of HA influenza (11–14), the existing prediction model research is still limited. Additionally, an increasing number of studies (15–17) indicated that ML algorithms possess numerous advantages in model construction. Based on the routine data of hospital admission, this study aims to explore the feature factors of HA influenza. By comparing the performance of multiple ML prediction models, we dedicate to constructing the optimal model and develop a practical prediction tool for early screening of HA influenza. This initiative aims to serve as a guide for monitoring HA influenza within healthcare facilities.

## Materials

### Study design

A retrospective, observational, single-centre study was conducted in Zhengzhou Central Hospital Affiliated to Zhengzhou University from January 2021 to May 2024. The sample consisted of patients aged 18 years and older, who had hospitalized for more than 7 days. The case group consisted of HA influenza patients, and the control group consisted of non-HA influenza patients who were admitted to the same ward in the HA influenza unit within 2 weeks. Finally, a total of 953 eligible subjects were included. Clinical information’s of subjects were collected through the hospital infection real-time monitoring system, hospital information system (HIS), and Laboratory Information System (LIS).

### Patient selection

Case group inclusion criteria: (a) HA influenza cases diagnosed 7 days or more after admission with no evidence of influenza infection at the time of admission, (b) HA influenza cases with positive PCR results, (c) HA influenza cases meeting the diagnostic criteria for hospital-acquired infections (18) who were admitted to the hospital for more than 48 h. Control group inclusion criteria: patients who were admitted to the same ward in the HA influenza unit within 2 weeks (1 week before or 1 week after). Exclusion criteria: (a) patients with missing data and duplicate data, (b) patients’ hospitalization≤7 days, or (c) patients age < 18 years.

## Methods

### Predictor variables

Information on patients with HA influenza was identified through the China Disease Control and Prevention Information System (CDCIS) and the Nosocomial infection surveillance system (NISS), and the HIS system retrieved and retrospectively analyzed the clinical data of all subjects. Specific inclusion data included information on gender, age, underlying diseases (hypertension, diabetes mellitus, chronic obstructive pulmonary disease, coronary heart disease, chronic renal failure), malignant tumors, immunosuppression, hematological disorders, cerebrovascular disorders, autoimmune disorders, lymphopenia, pregnancy, pneumonia on admission, glucocorticoid application, nutritional risk screening (NRS) score, and admission to ICU. Laboratory indicators include: white blood cell count, neutrophil count, procalcitonin, erythrocyte sedimentation rate, platelet count. Nutritional risk screening was conducted according to the NRS-2002 Nutritional Risk Screening scale. The test and examination data were derived from the first 48 h after the patient’s admission to the hospital.

### Calculation of sample size

The study involved 24 risk factors. According to EPP Principle (19), 5–10 positive patients were required for each risk factor in the modeling set. The number of positive patients should be between 120 and 240. Considering selection bias, the control group was selected for patients admitted to the same ward in the same ward of HA influenza within 2 weeks, which made it impossible to use EPP principle for reference estimation. The study indicated that the number of patients admitted to the same ward in the HA influenza unit within 2 weeks (1 week before or 1 week after) is 1–5 times higher than HA influenza patients, resulting in a maximum total sample size of 1,434. Larger sample sizes enhanced the generalization ability of predictive models. Consequently, the available data sample sizes in this study were 953.

### Model construction and evaluation

#### Feature factors screening

In this study, feature strategies of the wrapper-based Random Forest Boruta algorithm and the embedded Lasso regression technique were employed. The optimal subset determined by the two methods was considered as the key factors.

#### ML model construction and development

A variety of ML algorithm models were used for comprehensive analysis, and the optimal model was selected and constructed. The details were as follows:

Data set partitioning: To construct the predictive model, the dataset was randomly split into a 70% training subset and a 30% test subset. In the stage of model training, bootstrap resampling technique (a 5-fold cross-validation method) was used to optimize the model parameters and prevent the occurrence of model overfitting. The training set was randomly divided into five groups. Four groups were randomly selected for training in each iteration of the five-fold cross-validation as the training set, and the remaining group was considered as the validation set. In the stage of model assessment, the test set was used to evaluate the predictive performance of the model.

Selection of classification algorithm: Eight ML algorithm models were used for comprehensive analysis to compare the importance of each index in the training and validation sets of different models. The construction methods of prediction model include extreme gradient boosting (XGBoost), logistic regression (LR), light gradient boosting machine (LightGBM), random forest (RF), adaptive boosting (AdaBoost), support vector machine (SVM), k-nearest neighbors (KNN), and gaussian naive bayes (GNB).

Model training: Grid search method was used for constant adjusting to get optimal hyperparameters, models were retrained on the entire training set to derive the final model. Parameter values for ML models are shown in Supplementary Table 1.

Performance index: AUC value, accuracy, sensitivity, specificity, positive predictive value, negative predictive value, and F1 score.

Model comparison: The ROC comparison of each model was performed using DeLong test.

Considering performance indexes, we used the receiver operating characteristics (ROC) curve, calibration curve and precision-recall (PR) curve to evaluate the predictive performance of the models. The optimal model was finally screened. ROC curves were employed to assess the diagnostic efficacy of the model in the training set and validation set. A calibration curve was then plotted to evaluate the predictive effectiveness of the model. Learning curves were employed to evaluate the model’s fit and stability in the training and validation sets. Decision curve analysis (DCA) was used to assess the predictive efficiency and clinical applicability of the model.

### SHAP interpretability analysis

After the key factors have been identified, the significance of those were evaluated using the SHapley’s Additive Interpretation (SHAP) approach. The SHAP is a technique employed to interpret predictions generated by ML models, especially those that are complex and consist of a large number of features (20). The fundamental principle involved the computation of the incremental impact of individual features on the model’s output, enabling interpretation of the model’s behavior at both a global and local scale. Features with higher absolute SHAP values were identified as the most closely aligned with the model’s predictive scores.

### Statistical analysis

Continuous variables were expressed as mean ± standard deviation or median ± interquartile range and were analyzed using the unpaired *t* test or Mann–Whitney U test. Categorical variables were expressed as numbers and percentages and analyzed using the Chi-square test or Fisher exact test. Differences with *p* < 0.05 were considered statistically significant. Statistical software used included R (version 4.2.2), and Python (version 3.7).

The construction and evaluation of the models were carried out using Python 3.7 with package “xgboost 1.2.1” for xgboost, package “lightgbm 3.2.1” for lightgbm, and package “sklearn 0.22.1” for the remaining models. ROC curves, PR curves, and learning curves were plotted using the “sklearn 0.22.1” package, and SHAP analyses were performed using the “shap 0.39.0”package. LASSO regression analysis was performed using the glmnet package (version 4.1.7) in R, and the Boruta algorithm was applied using Boruta (version 8.0.0) in R. Similarly, the online prediction tool was constructed based on Shiny package in R.

## Results

### Demographic and clinical characteristics

A total of 5,063 patients with influenza were monitored, and 239 patients with HA influenza, representing 4.7% of the total. Of the total number of cases, 112 were male and 127 were female. The mean age of the patients was 46.23 ± 11.21 years. Among the HA influenza subtypes, influenza A accounted for 63.4%, influenza B accounted for 26.4% and unclear classification accounted for 10.2%. The top five departments in terms of proportion are respiratory (30/12.64%), ICU (28/11.72%), nephrology (22/9.21%), hematology (18/7.53%) and urology (15/6.28%). Furthermore, 118 cases of HA influenza were documented in real-time hospital infection surveillance system, representing only 49.4% of cases were reported.

### Comparison of baseline characteristics

The baseline characteristics for the case group and control group were shown in Table 1. Compared to the control group, patients with HA influenza were more likely to be older and to have a higher BMI or nutritional risk. There were more patients diagnosed with pneumonia, chronic kidney failure, malignancy, hypoproteinemia, autoimmune disease, and lymphocytopenia on admission in the case group. At the same time, a considerable number of patients in the case group had been admitted to ICU. In addition, laboratory-related factors were not statistically significant between the two groups (*p* > 0.05).

**Table 1:** Comparison of baseline characteristics between the case group and control group.

Factors	Missing data	Category	Total (*n* = 953)	Control (*n* = 714)	Case (*n* = 239)	Statistic	p*
Age (years) (%)	0 (0%)	<60	724 (76)	564 (79)	160 (67)	14.233	<0.001
		≥60	229 (24)	150 (21)	79 (33)		
Gender (%)	0 (0%)	Female	292 (30.6)	226 (31.7)	66 (27.6)	1.374	0.241
		Male	661 (69.4)	488 (68.3)	173 (72.4)		
Pneumonia on admission (%)	0 (0%)	No	832 (87.3)	644 (90.2)	188 (78.7)	21.494	<0.001
		Yes	121 (12.7)	70 (9.8)	51 (21.3)		
Hypertension (%)	0 (0%)	No	292 (30.6)	207 (29.0)	85 (35.6)	3.641	0.056
		Yes	661 (69.4)	507 (71.0)	154 (64.4)		
Diabetes (%)	0 (0%)	No	592 (62.1)	444 (62.2)	148 (61.9)	0.005	0.943
		Yes	361 (37.9)	270 (37.8)	91 (38.1)		
COPD (%)	0 (0%)	No	871 (91.4)	652 (91.3)	219 (91.6)	0.023	0.880
		Yes	82 (8.6)	62 (8.7)	20 (8.4)		
CHD (%)	0 (0%)	No	822 (86.3)	617 (86.4)	205 (85.8)	0.062	0.803
		Yes	131 (13.7)	97 (13.6)	34 (14.2)		
CRF (%)	0 (0%)	No	829 (87)	637 (89.2)	192 (80.3)	12.478	<0.001
		Yes	124 (13)	77 (10.8)	47 (19.7)		
MT (%)	0 (0%)	No	868 (91.1)	664 (93)	204 (85.4)	12.871	<0.001
		Yes	85 (8.9)	50 (7)	35 (14.6)		
Hypoproteinemia (%)	0 (0%)	No	837 (87.8)	645 (90.3)	192 (80.3)	16.754	<0.001
		Yes	116 (12.2)	69 (9.7)	47 (19.7)		
CVD (%)	0 (0%)	No	882 (92.5)	661 (92.6)	221 (92.5)	0.003	0.956
		Yes	71 (7.5)	53 (7.4)	18 (7.5)		
AD (%)	0 (0%)	No	928 (97.4)	702 (98.3)	226 (94.6)	9.903	0.002
		Yes	25 (2.6)	12 (1.7)	13 (5.4)		
Pregnancy (%)	0 (0%)	No	906 (95.1)	674 (94.4)	232 (97.1)	2.729	0.099
		Yes	47 (4.9)	40 (5.6)	7 (2.9)		
Glucocorticoid use (%)	0 (0%)	No	816 (85.6)	628 (88)	188 (78.7)	12.566	<0.001
		Yes	137 (14.4)	86 (12)	51 (21.3)		
NRS (%)	0 (0%)	<3	771 (80.9)	598 (83.8)	173 (72.4)	14.979	<0.001
		≥3	182 (19.1)	116 (16.2)	66 (27.6)		
Hemopathy (%)	0 (0%)	No	921 (96.6)	693 (97.1)	228 (95.4)	1.523	0.217
		Yes	32 (3.4)	21 (2.9)	11 (4.6)		
Admission to ICU (%)	0 (0%)	No	864 (90.7)	662 (92.7)	202 (84.5)	14.214	<0.001
		Yes	89 (9.3)	52 (7.3)	37 (15.5)		
Lymphopenia (%)	0 (0%)	No	917 (96.2)	699 (97.9)	218 (91.2)	22.020	<0.001
		Yes	36 (3.8)	15 (2.1)	21 (8.8)		
BMI (kg/m^2^) (IQR)	0 (0%)		25.712 (23.875, 27.548)	25.528 (23.459, 27.344)	25.952 (24.382, 27.778)	−3.174	0.002
PCT (μg/L) (IQR)	0 (0%)		0.235 (0.200, 0.263)	0.238 (0.193, 0.263)	0.230 (0.205, 0.263)	0.352	0.725
WBC count (*10^9^/L)(IQR)	0 (0%)		7.050 (6.250, 8.371)	7.050 (6.270, 8.300)	7.000 (5.970, 8.580)	0.521	0.602
ESR (mm/h) (IQR)	0 (0%)		13.000 (6.000, 29.000)	12.000 (6.000, 29.000)	14.000 (6.000, 25.000)	−0.505	0.613
NEUT count (*10^9^/L) (IQR)	0 (0%)		4.360 (3.460, 6.040)	4.298 (3.460, 5.988)	4.470 (3.510, 6.079)	−0.552	0.581
PLT count (*10^9^/L) (IQR)	0 (0%)		212.000 (173.000, 249.000)	216.000 (176.000, 249.000)	201.000 (164.000, 245.000)	1.827	0.068

* *p* value < 0.05 was considered significant. The statistics were obtained by Mann Whitney-U test or Chi-square test. Data were shown as number (percentage) or median (IQR, interquartile range). COPD, chronic obstructive pulmonary disease; CHD, coronary heart disease; CRF, chronic renal failure; MT, malignant tumor; CVD, cerebrovascular disease; AD, autoimmune disease; NRS, nutritional risk screening; ESR, erythrocyte sedimentation rate; PLT, platelet; WBC, white blood cell; PCT, procalcitonin; NEUT, neutrophil.

### Feature selection

A total of 953 patients were divided into 667 cases in the training group and 286 cases in the testing group in the ratio of 7:3. Statistical analysis showed no significant difference was between the two groups (all *p* > 0.05), as shown in Supplementary Table 2.

The Boruta algorithm (an extension of the RF algorithm) was utilized to identify the actual set of features by accurately estimating the significance of each feature (21). The Boruta algorithm identified 19 key factors including age, gender, BMI, pneumonia on admission, diabetes, COPD, CHD, CRF, MT, hypoproteinemia, CVD, AD, etc. In contrast, variables were analyzed by LASSO regression that can compress variable coefficients to prevent overfitting and solve serious covariance problems (22). The results showed that 24 independent factors were screened and finally simplified to 10 key factors, namely age, BMI, CRF, MT, CVD, pneumonia on admission, lymphopenia, hypoproteinemia, glucocorticoid use, admission to ICU.

**Figure 1:**
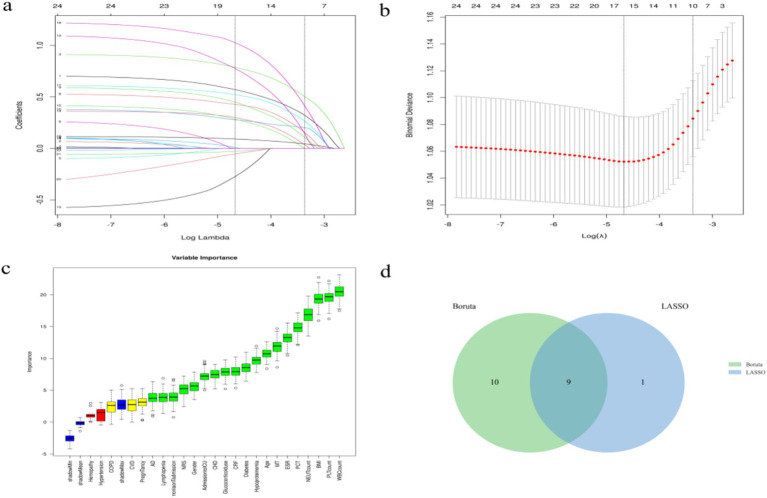
Screening process of feature variables from LASSO regression analysis and Boruta algorithm. **(a,b)** Factor screening based on the LASSO regression model, with the left dashed line indicating the best lambda value for the evaluation metrics (lambda.min) and the right dashed line indicating the lambda value for the model where the evaluation metrics are in the range of the best value by one standard error (lambda.1se); **(c)** Boruta algorithm screening variable trajectories; **(d)** The common subset of Boruta and LASSO.

By the screening results from the LASSO regression and the Boruta algorithm, we identified a common subset of key factors selected by both methods (Figure 1). Finally, age, pneumonia on admission, CRF, MT, hypoproteinemia, glucocorticoid use, admission to ICU, lymphopenia, BMI were identified as feature factors used for model construction.

### Comparison of multiple classification models

**Figure 2:**
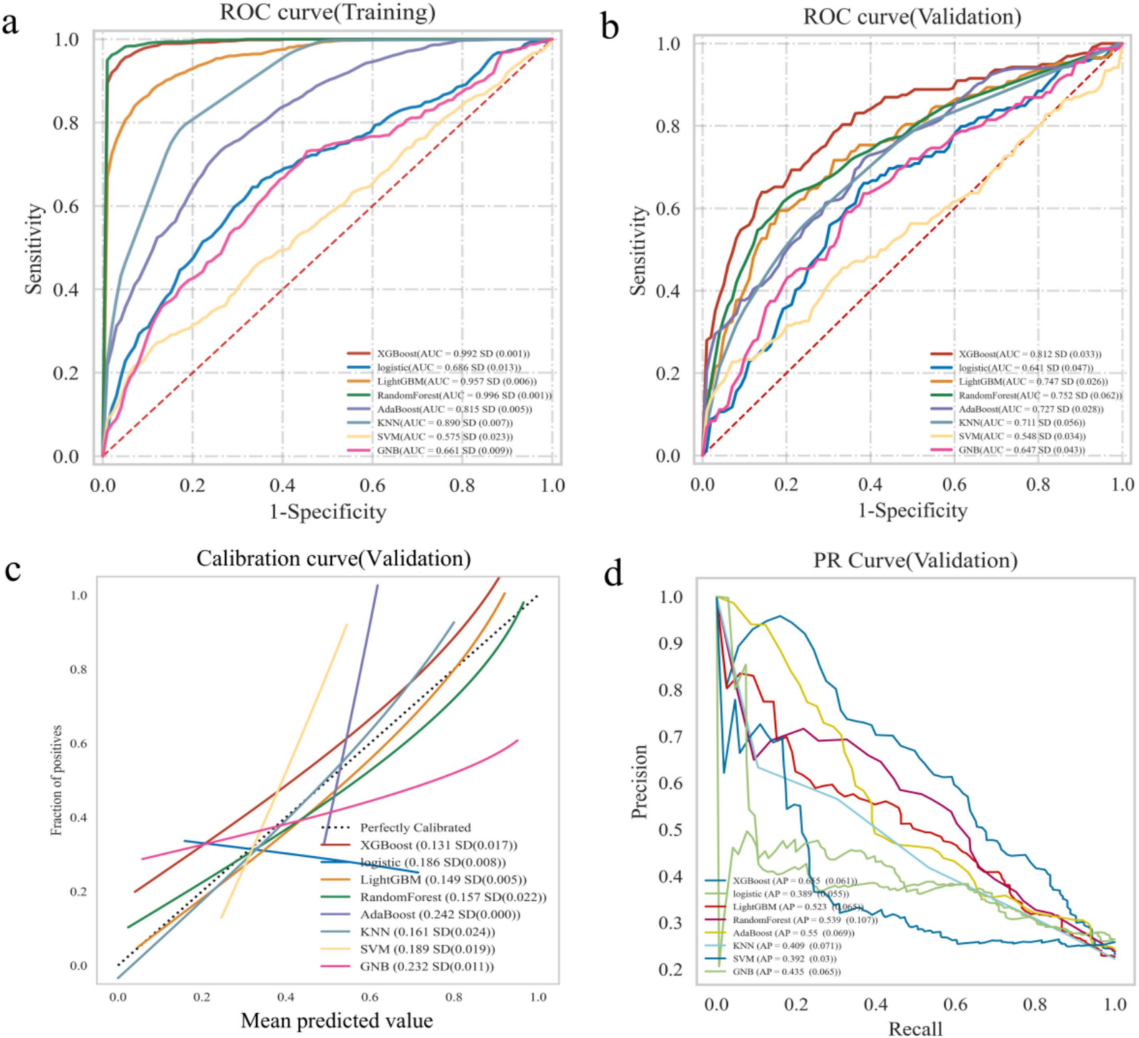
Construction and comparison of multiple ML algorithms models. **(a)** The ROC curve analysis in training set. **(b)** The ROC curve analysis in validation set. **(c)** Calibration curve of ML models in validation sets. **(d)** PR curves of ML models in validation sets.

The XGBoost, LR, LightGBM, RF, AdaBoost, SVM, KNN and GNB models were trained and validated. The models were evaluated using AUC values (23), which demonstrated that RF exhibited the highest performance in the training set, with AUC value of 0.996 and F1 score of 0.960. While XGBoost demonstrated the highest performance in the validation set, with AUC value of 0.812 and F1 score of 0.590 (Table 2). The AUC values focused on the predictive accuracy of the models and failed to more effectively filter optimal models. Consequently, calibration curves and the area under the PR curve were examined. The calibration curves in the validation set demonstrated the highest accuracy of XGBoost model, accompanied by the highest AP value of 0.655 (Figure 2). The results obtained from the training and validation sets suggested that the RF model might be overfitting, while the XGBoost model exhibited relatively greater stability on the validation set. A comprehensive analysis further indicated that the XGBoost model demonstrated the most optimal performance across all evaluated metrics.

**Table 2:** Predictive performance of eight ML algorithms in the training and validation sets of the HA influenza screening model.

Classification models	AUC	Cutoff	Accuracy	Sensitivity	Specificity	Positive predictive value	Negative predictive value	F1 scoring
Training set								
XGBoost	0.992	0.300	0.958	0.955	0.959	0.887	0.985	0.920
LR	0.686	0.235	0.674	0.647	0.683	0.403	0.857	0.494
LightGBM	0.957	0.315	0.896	0.878	0.902	0.758	0.957	0.811
RF	0.996	0.435	0.980	0.961	0.986	0.960	0.987	0.960
AdaBoost	0.815	0.494	0.735	0.747	0.732	0.480	0.897	0.584
KNN	0.890	0.400	0.824	0.778	0.839	0.623	0.918	0.691
SVM	0.575	0.254	0.761	0.247	0.927	0.530	0.792	0.331
GNB	0.661	0.090	0.614	0.715	0.582	0.356	0.864	0.474
Validation set								
XGBoost	0.812	0.300	0.800	0.622	0.852	0.573	0.883	0.590
LR	0.641	0.235	0.640	0.561	0.668	0.373	0.815	0.445
LightGBM	0.747	0.315	0.772	0.575	0.829	0.496	0.870	0.532
RF	0.752	0.435	0.787	0.502	0.875	0.553	0.850	0.525
AdaBoost	0.727	0.494	0.682	0.641	0.693	0.399	0.861	0.489
KNN	0.711	0.400	0.730	0.532	0.787	0.418	0.854	0.466
SVM	0.548	0.254	0.740	0.201	0.930	0.505	0.770	0.281
GNB	0.647	0.090	0.609	0.680	0.582	0.372	0.836	0.479

### Construction and evaluation of the optimal model

**Figure 3:**
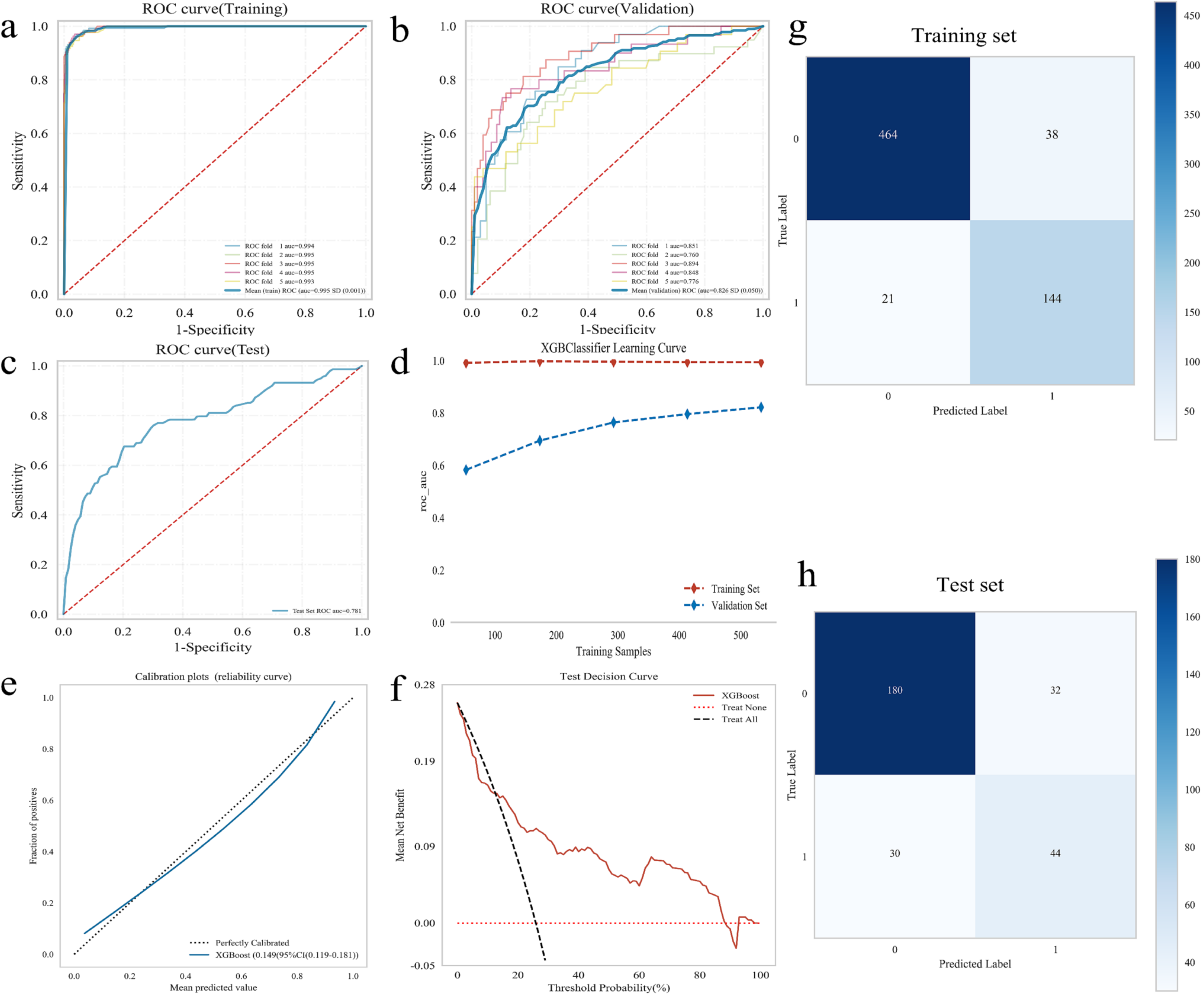
Construction and evaluation of XGBoost model. **(a–c)** ROC curve, including training set **(a)**, validation set **(b)**, and test set **(c)**; **(d)** XGBoost classifier learning curve; **(e)** Calibration curve of the model; **(f)** DCA diagram of the model; **(g)** Confounding matrix for the training set; **(h)** Confounding matrix for the test set.

A 5-fold cross-validation was performed on the training set. The results indicated that the average AUC value for the training set was 0.995, while the average AUC value for the validation set was 0.826. Additionally, the AUC value of the test set was 0.781 (Figures 3a-c). The AUC values of the training set, validation set, and test set eventually stabilized around 0.8, demonstrating accurate model predictions. When the performance of the validation set under the AUC metric is lower than that of the test set or the ratio is less than 10%, the model can be considered successfully fitted (24). The learning curves suggested that the training and validation sets exhibit strong fitting capabilities and high stability (Figure 3d). The calibration curve confirmed the model’s good accuracy and discriminative ability, while the decision curve analysis demonstrated that the predictive model got high predictive value and clinical significance (Figures 3e,f). Furthermore, the confusion matrix results revealed differences in the model’s performance across different datasets. In the training set (Figure 3g), the true positive rate (sensitivity) was 96.1%, and the true negative rate (specificity) was 96.4%. In the test set (Figure 3h), the true positive rate was 59.5%, and the true negative rate was 84.9%. These findings indicated that the XGBoost model is fully applicable for classification modeling of the dataset.

### Model interpretation

**Figure 4:**
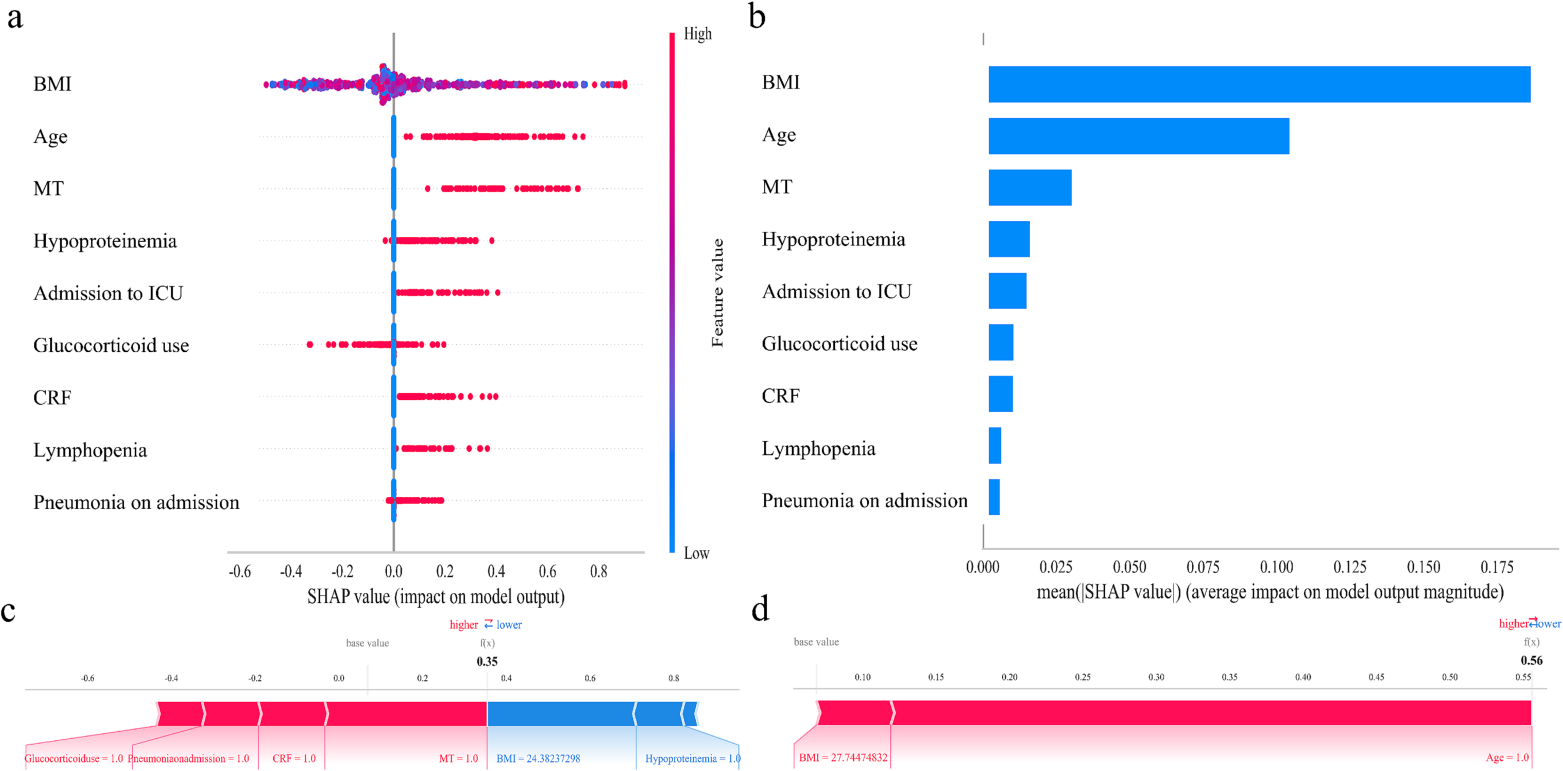
SHAP analysis of the XGBoost model. **(a)** SHAP dendrogram of features. **(b)** Importance ranking plot of features. **(c,d)** Interpretability analysis of 2 independent samples.

Initially, 24 independent variables were screened and finally simplified to 9. We used SHAP analysis to visualize the interpretation of feature factors. 9 most important features in our model were showed in Figure 4a. Within each feature significance line, the attribution of all patients to the outcome was plotted with dots of different colors, where red dots indicated high risk values and blue dots indicated low risk values. Patients with increased BMI, age (>60 years), pneumonia on admission, ICU admission, glucocorticoid use, presence of chronic renal failure, lymphopenia, malignant tumor or hypoproteinemia were at high risk for HA influenza.

Figure 4b shows the ranking of the 9 feature factors assessed by mean absolute SHAP values, with the X-axis SHAP values indicating the importance of the predictive model. In addition, we provided two typical examples to illustrate the interpretability of the model. For each patient, the model generates a predictive value, expressed as a SHAP score, which quantifies individual risk. A patient with a relatively low SHAP score of 0.35 (Figure 4c) is at a low risk of HA influenza. In contrast, another patient with a significantly higher SHAP score of 0.56 (Figure 4d) faces a high risk of HA influenza.

### Model presentation

**Figure 5:**
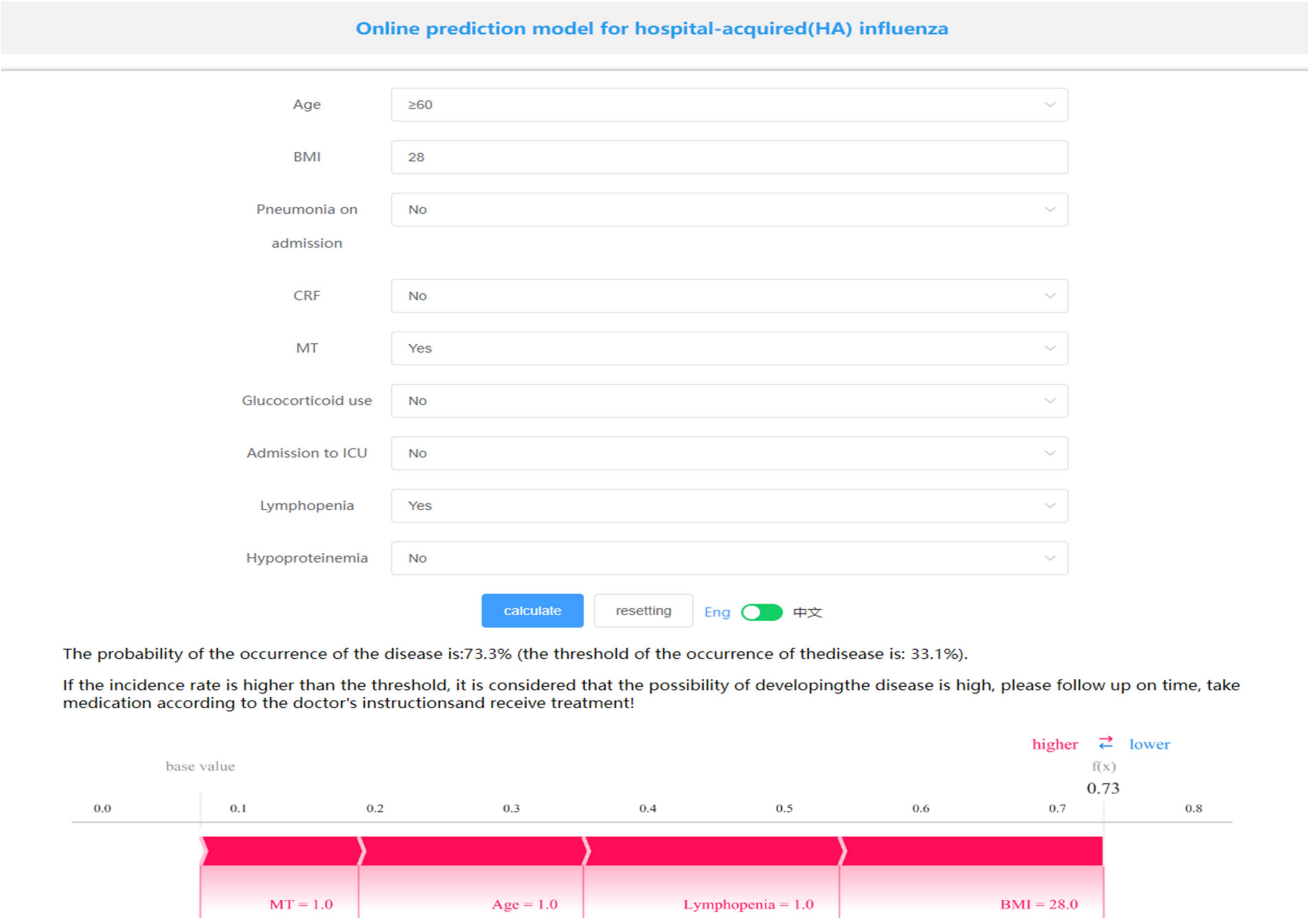
Online prediction model for HA influenza and individual patient risk presentation.

A visualization and online prediction model was constructed at http://www.xsmartanalysis.com/model/list/predict/model/html?mid=23476&symbol=2Hb17hd417409jS1AR84, researchers can analyze and verify the performance of the model online. A screenshot of the presentation of the generic model is shown in Figure 5. A Patient aged ≥60 years, with a BMI of 28, a malignant tumor, and lymphopenia, have a 73.3% probability of developing HA influenza, placing them in the high-risk group. Early prevention measures and timely interventions should be implemented to mitigate this risk.

## Discussion

It has been proposed that standardized surveillance of HA influenza and effective establishment of a respiratory protection system can reduce the infection and mortality rates of HA influenza (6). In this study, HA influenza patients accounted for about 5% of all influenza patients. However, according to the Real-Time Hospital Infection Surveillance System, only about 50% of the HA influenza cases were documented, indicating that nearly half of the cases were not officially reported. This also highlights the critical importance of standardized HA influenza surveillance in hospital governance. Although the system is essential, assessing its effectiveness and achieving optimal results remains challenging. In our study, we developed and validated several widely-used machine learning (ML) algorithms, constructed an HA influenza prediction model using routinely collected data, and created a quantifiable, online prediction tool. This facilitates early intervention, thereby reducing the infection rate and lowering the morbidity and mortality rates associated with HA influenza.

This study employed two feature selection methods to screen out 9 feature factors including age, pneumonia on admission, CRF, MT, hypoproteinemia, glucocorticoid use, admission to ICU, lymphopenia, BMI from 24 clinical variables. Several studies (25, 26) have identified age (≥65 years) and presence of underlying disease to be important characteristics of HA influenza. Meanwhile, a case–control study conducted in a Chinese population demonstrated that lymphopenia, hypoproteinemia, and pleural effusion serve as independent risk factors for patients at high risk of HA influenza A (27). Additionally, a large cross-sectional study (12) analyzed the features of HA influenza over a 10-year period and revealed that immunodeficiency, ICU admission, recurrent bacterial infections, and respiratory distress were strongly correlated with HA influenza when compared to community-acquired influenza. Similarly, another study confirmed a significant association between HA influenza and increased hospitalization rates as well as in-hospital mortality in intensive care units (ICUs) (28). Furthermore, hypoalbuminemia, which frequently arises from a combination of inflammation and insufficient protein and caloric intake in patients with chronic conditions such as chronic renal failure, is strongly associated with the development of HA influenza.

Machine learning is a method that leverages data to train a model and then uses the model to make predictions, mainly including supervised learning, unsupervised learning, and reinforcement learning. Compared with classical statistical regression models, ML algorithms exhibit numerous advantages, such as being less constrained by strict assumptions regarding variable distributions and numbers, as well as demonstrating greater robustness to missing data (29). XGBoost can efficiently deal with missing data and construct accurate predictive models (30). LightGBM demonstrates outstanding performance when processing extremely large structured datasets, featuring exceptionally high training speed. However, its performance is sensitive to the number of features and sample size (31). Random Forest (RF) achieves high classification accuracy but demands substantial computational resources (32). Another example is the TAN Bayesian network, which effectively utilizes all variables and their interaction information to depict the conditional dependency network between the dependent variable and predictor variables. As variable information increases, the conditional probabilities among independent variables are dynamically updated via reverse inference, enabling real-time model adjustment and enhancing prediction efficacy (33). Using four ML algorithms to construct a prediction model for HA influenza, a study found that the random forest model performed the best in predicting HA influenza, with an AUC of 83.3%, and also pointed out that living in a double room was the most important predictor of HA influenza (34).

In feature selecting, univariable selection methods are generally not recommended because they fail to account for the association between predictors and could lead to loss of valuable information. Actually, selecting only features that exhibit apparent linear interactions for feature selection prior to machine learning (ML) training inherently possesses certain limitations. Some existing feature selection methods include filter methods, wrapper methods, embedded methods, etc. Nonlinear feature selection methods, such as random forest model, can automatically capture nonlinear relationships in data. The complex relationship between features and target variables is found by constructing decision tree structure through recursive partition of features. While for small sample data sets, wrapper methods (recursive feature elimination, RFE) or embedded methods (Lasso regression) often yield superior outcomes by integrating model performance during feature selection. Ideally, an prediction model should incorporate multiple ML algorithms and be optimized according to specific clinical requirements. The model should possess good generalizability, high predictive efficacy, strong adaptability and practicality. Additionally, it should be validated by a multicenter large-sample prospective clinical cohort study.

Despite the results that some published studies selected community-acquired influenza (CAI) patients as controls (25), our study selected controls who were hospitalized in the same department and during the same time period without acquiring HA influenza, thus the comparability between the case group and the control group can be ensured. Furthermore, as recommended by the BMJ Predictive Model Guidelines (35), valid internal validation is more reliable than a meaningless and misleading external validation. To be exact, more rigorous internal validation was performed in this study.

However, our study has some limitations. First, the sample size of this study was relatively small and the data were obtained from a single institution rather than a multicenter study. Therefore, the generalizability of these findings is limited. And despite restrictions to control selection bias and the high degree of consistency achieved in the reproducibility analyses of the training and test sets, some unavoidable bias may still occur due to the uncertainty of segmentation. In addition, certain indicators, such as influenza vaccination status, were not included in the analysis. Longitudinal or prospective case–control studies are necessary to further elucidate the relationship between HA influenza and risk factors. Although this study employs eight ML methods, the emergence of the Tabular Prior-data FittedNetwork (TabPFN) (36) compels us to reassess and validate predictive performance of traditional ML models.

## Conclusion

In summary, this study aimed to construct a prediction model based on multiple ML algorithms, with the XGBoost model demonstrating superior performance. Additionally, we successfully developed a simple, practical and personalized online risk assessment tool. Developing a screening model can effectively assist clinicians in formulating more precise prevention and treatment strategies, as well as identifying and intervening in the occurrence of HA influenza. The subsequent step will involve integrating additional data to enhance the performance of model. This also necessitates conducting more extensive research and involving a broader population to further validate the model’s performance.

## Data Availability

The original contributions presented in the study are included in the article/Supplementary material, further inquiries can be directed to the corresponding author.
