# Supplementary material for: Constructing a screening model to identify patients at high risk of hospital-acquired influenza on admission to hospital
Source: Front Public Health. 2025 Apr 16;13:1495794. doi: 10.3389/fpubh.2025.1495794 (PMC12041216; doi:10.3389/fpubh.2025.1495794)
Supplement: Supplementary file 1 [file Table_1.DOCX]

Parameter values for ML models

| Clssification models | Model parameters |
| --- | --- |
| XGBoost | reg_lambda : 0.5 min_child_weight : 2 max_depth : 8 learning_rate : 0.3 |
| Logistic | tol : 0.0001 penalty : l2 max_iter : 100 C: 1.0 |
| LightGBM | num_leaves: 100 n_estimators : 50 max_depth : 20 learning_rate : 0.001 boosting_type : gbdt |
| RandomForest | n_estimators : 100 min_impurity_decrease : 0.0 max_depth : None criterion : gini |
| AdaBoost | n_estimators : 50 learning_rate : 1.0 |
| KNN | Weights : distance n_neighbors : 2 |
| GNB | var_smoothing : 1e-07 |
| SVM | tol : 0.001 kernel : rbf C : 1.0 |
