# Supplementary material for: Constructing a screening model to identify patients at high risk of hospital-acquired influenza on admission to hospital
Source: Front Public Health. 2025 Apr 16;13:1495794. doi: 10.3389/fpubh.2025.1495794 (PMC12041216; doi:10.3389/fpubh.2025.1495794)
Supplement: Supplementary file 2 [file Table_2.DOCX]

Supplementary Table 2 Comparison of baseline characteristics between the training set and test set

| Factors | Missing data | Category | Total  (n=953) | Training set (n=667) | Test set  (n=286) | Statistic | *P** |
| --- | --- | --- | --- | --- | --- | --- | --- |
| Age (years) (%) | 0 (0%) | ＜60 | 660(69.3) | 459(68.8) | 201(70.3) | 0.202 | 0.653 |
|  |  | ≥60 | 293(30.7) | 208(31.2) | 85(29.7) |  |  |
| Gender (%) | 0 (0%) | female | 283(29.7) | 200(30.0) | 83(29.0) | 0.089 | 0.765 |
|  |  | Male | 670(70.3) | 467(70.0) | 203(71.0) |  |  |
| Pneumonia on admission (%) | 0 (0%) | No | 800(83.9) | 562(84.3) | 238(83.2) | 0.161 | 0.688 |
|  |  | Yes | 153(16.1) | 105(15.7) | 48(16.8) |  |  |
| Hypertension (%) | 0 (0%) | No | 657(68.9) | 463(69.4) | 194(67.8) | 0.234 | 0.628 |
|  |  | Yes | 296(31.1) | 204(30.6) | 92(32.2) |  |  |
| Diabetes (%) | 0 (0%) | No | 619(65) | 431(64.6) | 188(65.7) | 0.110 | 0.741 |
|  |  | Yes | 334(35) | 236(35.4) | 98(34.3) |  |  |
| COPD(%) | 0 (0%) | No | 872(91.5) | 612(91.8) | 260(90.9) | 0.184 | 0.668 |
|  |  | Yes | 81(8.5) | 55(8.2) | 26(9.1) |  |  |
| CHD(%) | 0 (0%) | No | 820(86) | 575(86.2) | 245(85.7) | 0.049 | 0.825 |
|  |  | Yes | 133(14) | 92(13.8) | 41(14.3) |  |  |
| CRF(%) | 0 (0%) | No | 853(89.5) | 595(89.2) | 258(90.2) | 0.215 | 0.643 |
|  |  | Yes | 100(10.5) | 72(10.8) | 28(9.8) |  |  |
| MT(%) | 0 (0%) | No | 883(92.7) | 616(92.4) | 267(93.4) | 0.296 | 0.587 |
|  |  | Yes | 70(7.3) | 51(7.6) | 19(6.6) |  |  |
| Hypoproteinemia(%) | 0 (0%) | No | 822(86.3) | 573(85.9) | 249(87) | 0.226 | 0.635 |
|  |  | Yes | 131(13.7) | 94(14.1) | 37(13) |  |  |
| CVD(%) | 0 (0%) | No | 888(93.2) | 626(93.9) | 262(91.6) | 1.587 | 0.208 |
|  |  | Yes | 65(6.8) | 41(6.1) | 24(8.4) |  |  |
| AD(%) | 0 (0%) | No | 924(97) | 648(97.2) | 276(96.5) | 0.285 | 0.594 |
|  |  | Yes | 29(3) | 19(2.8) | 10(3.5) |  |  |
| Pregnancy(%) | 0 (0%) | No | 918(96.3) | 644(96.6) | 274(95.8) | 0.316 | 0.574 |
|  |  | Yes | 35(3.7) | 23(3.4) | 12(4.2) |  |  |
| Glucocorticoid use(%) | 0 (0%) | No | 801(84.1) | 562(84.3) | 239(83.6) | 0.071 | 0.789 |
|  |  | Yes | 152(15.9) | 105(15.7) | 47(16.4) |  |  |
| NRS(%) | 0 (0%) | ＜3 | 776(81.4) | 541(81.1) | 235(82.2) | 0.148 | 0.700 |
|  |  | ≥3 | 177(18.6) | 126(18.9) | 51(17.8) |  |  |
| Hemopathy(%) | 0 (0%) | No | 926(97.2) | 652(97.8) | 274(95.8) | 2.756 | 0.097 |
|  |  | Yes | 27(2.8) | 15(2.2) | 12(4.2) |  |  |
| Admission to ICU(%) | 0 (0%) | No | 843(88.5) | 590(88.5) | 253(88.5) | 0.000 | 0.998 |
|  |  | Yes | 110(11.5) | 77(11.5) | 33(11.5) |  |  |
| Lymphopenia(%) | 0 (0%) | No | 910(95.5) | 637(95.5) | 273(95.5) | 0.001 | 0.974 |
|  |  | Yes | 43(4.5) | 30(4.5) | 13(4.5) |  |  |
| BMI(kg/m^2^) (IQR) | 0 (0%) |  | 25.712[23.875,27.548] | 25.712[23.758,27.548] | 25.867[24.183,27.548] | -1.146 | 0.252 |
| PCT(μg/L) (IQR) | 0 (0%) |  | 0.240[0.200,0.270] | 0.240[0.200,0.270] | 0.240[0.210,0.270] | -0.832 | 0.405 |
| WBC count (*10^9^/L)(IQR) | 0 (0%) |  | 7.050[6.240,8.390] | 7.070[6.250,8.410] | 6.970[6.190,8.280] | 0.802 | 0.423 |
| ESR(mm/h) (IQR) | 0 (0%) |  | 13.000[5.000,27.000] | 13.000[5.000,27.000] | 13.000[5.000,30.000] | -0.458 | 0.646 |
| NEUT count (*10^9^/L) (IQR) | 0 (0%) |  | 4.250[3.370,5.840] | 4.300[3.400,5.850] | 4.170[3.360,5.780] | 0.817 | 0.414 |
| PLT count(*10^9^/L) (IQR) | 0 (0%) |  | 208.000[174.000,247.000] | 210.000[173.000,247.000] | 205.000[174.000,248.000] | 0.620 | 0.536 |
